# Fabrication of fillable microparticles and other complex 3D microstructures

**DOI:** 10.1126/science.aaf7447

**Published:** 2017-09-15

**Authors:** Kevin J. McHugh, Thanh D. Nguyen, Allison R. Linehan, David Yang, Adam M. Behrens, Sviatlana Rose, Zachary L. Tochka, Stephany Y. Tzeng, James J. Norman, Aaron C. Anselmo, Xian Xu, Stephanie Tomasic, Matthew A. Taylor, Jennifer Lu, Rohiverth Guarecuco, Robert Langer, Ana Jaklenec

**Affiliations:** David H. Koch Institute for Integrative Cancer Research, Massachusetts Institute of Technology, Cambridge, MA 02139, USA

## Abstract

Three-dimensional (3D) microstructures created by microfabrication and additive manufacturing have demonstrated value across a number of fields, ranging from biomedicine to microelectronics. However, the techniques used to create these devices each have their own characteristic set of advantages and limitations with regards to resolution, material compatibility, and geometrical constraints that determine the types ofmicrostructures that can be formed.We describe a microfabrication method, termed StampEd Assembly of polymer Layers (SEAL), and create injectable pulsatile drug-delivery microparticles, pH sensors, and 3D microfluidic devices that we could not produce using traditional 3D printing. SEAL allows us to generate microstructures with complex geometry at high resolution, produce fully enclosed internal cavities containing a solid or liquid, and use potentially any thermoplastic material without processing additives.

Three-dimensional (3D) microstructures have potential use in a wide array of biomedical (tissue engineering and drug delivery),microelectromechanical (sensors and actuators), energy, and environmental applications (*1**–**4*). Although a number of extrusion, sintering, and light-based additive manufacturing processes (i.e., 3D printing) have been developed to create these devices, each of these methods has advantages and disadvantages that make it applicable to only a subset ofmicrostructures. Therefore, the most appropriate fabrication technique is typically selected by considering the size, shape, and composition of the desiredmicrodevice because each technique has limitations in spatial resolution, device geometry, material compatibility, and/ or throughput (*5*, *6*). For example, stereolithography and fused depositionmodeling, which have emerged as leaders in the field of custom manufacturing, are subject to a trade-off between resolution and the materials that can be printed (*7*). High-resolution stereolithographic 3D printing can produce nanoscale features but requires photoactive processing additives (some of which have unknown safety profiles in humans) and is not compatible with materials relevant for biomedical applications, such as poly(lactic-co-glycolic acid) (PLGA) and polycaprolactone (*8**–**10*). These processes also rely on liquid polymerization or cross-linking and may not be compatible with the encapsulation of drugs or other sensitivemolecules owing to the presence of the liquid prepolymer solution that could denature or solubilize the cargo. Alternatively, heat-based fused deposition modeling, although theoretically compatible with any thermoplastic polymer, lacks the control needed to create microstructures with high resolution (*11*). Single-step micromolding processes, such as particle replication in nonwetting templates (PRINT), are attractive for their nanoscale resolution and throughput. However, these approaches are limited to single-layer geometries that can be released from a mold, which makes it difficult to fabricate structures that have internal architecture or a “top-narrowing” 3D shape (*12*, *13*).

We have developed a bottom-up, high-resolution microstructure fabrication technique to create microdevices with complex geometries using a variety of commercially relevant materials, including lactide-glycolide copolymers, the most widely used biodegradable polymers for human applications. This approach, termed StampEd Assembly of polymer Layers (SEAL), combines the technology used for computer chip manufacturing with soft lithography and an aligned sintering process to produce small (≤400 µm) polymeric structures (movie S1 and fig. S1). Two or more silicon molds with complementary patterns are etched by standard microfabrication techniques (fig. S1, A to J). Polydimethylsiloxane (PDMS) is then cured on the surface of each silicon wafer to produce inverse elastomeric molds (fig. S1, K and L). A polymer is heated and pressed into the PDMS molds to produce the laminar microstructure components of interest. The first layer is then delaminated onto a separate surface, such as glass, by heat-assisted microtransfer molding. Subsequent layers of the final structure are assembled by a layer-by-layer sintering process under microscopic alignment to produce a large array ofmicrostructures (Fig. 1). This process draws on elements from existing technology, including laminated object manufacturing (*14*), microfabrication-based surface patterning (*15*), and thermal bonding of PLGA (*16*) to create discrete polymeric microdevices with well-defined geometry.

**Fig. 1:**
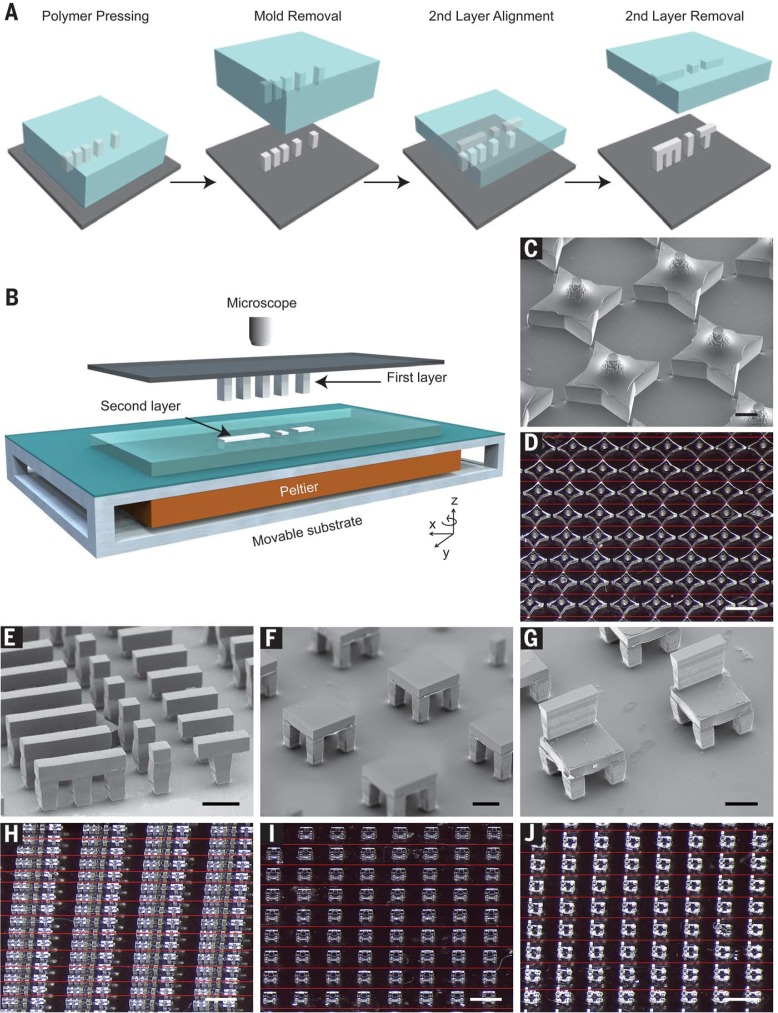
**Assembly of 3D microstructures using the SEAL process**. (**A**) Microstructures are fabricated by pressing and heating polymer into a patterned PDMS base mold and delaminating these structures onto a substrate to create the first layer. A second layer is then formed by using a similar molding process against a Teflon surface, which allows the features to remain in the PDMS mold after cooling. The second layer is aligned, placed into contact with the first layer, and sintered by using a mild heating step. (**B**) Schematic depicting the alignment and sintering equipment, consisting of a mask aligner retrofitted with a Peltier heater. A glass slide containing the first layer is suspended upside down from a fixed mask holder by means of a vacuum while the second layer, still in the PDMS mold, is placed on the wafer chuck, aligned using the stage rotation and translation knobs, put into contact, and heated until they fuse.This approach can be used to create a variety ofmicrostructures, including (**C** and **D**) stars, (**E** and **H**) letters spelling “MiT,” (**F** and **I**) two-layered tables, and (**G** and **J**) three-layered chairs. Scale bars indicate 200 mmfor scanning electron microscopy (SEM) images and 1mmfor optical images.Optical images were stitched together from multiple images to enable a better depth of focus.The interfaces between images are denoted by thin red lines.

To ensure high-fidelitymicrodevice fabrication, we have developed a technique to align layers during sintering with high precision. This approach uses a photomask aligner (MA4, Karl Suss, Sunnyvale, California) retrofitted with a Peltier heater, temperature controller, relay, and voltage source to enable simultaneous alignment and thermal bonding (Fig. 1B). The mask holder vacuumwas applied to hold a glass slide containing the first microstructure layer facing down while the next layer, still in the PDMS mold, was held on the wafer chuck. After optically aligning adjacent features with the mask aligner’s microscope and alignment knobs, the layers were brought into contact and heated to just above the polymer’s glass transition temperature (table S1) for up to 3 min. The sealing process was continuously monitored during this time by observing the disappearance of light diffraction patterns (movie S2). As two layers came into contact, the small air gap between themproduced diffraction that resolved when the heated polymer flowed to close the gap. After cooling samples to room temperature, the PDMS micromold containing the second layer was peeled off to yield a multilayered microstructure. Individual devices were then removed from the glass slide. This process was used to create large arrays of microstructures, including a 3Dstar, two-layered letters spelling “MiT,” a two-layer table with high– aspect-ratio supports, and a three-layer chair (Fig. 1, C to J). Mechanical characterization of the PLGA microstructures indicated that the adhesion strength of adjacent layers was of similar magnitude as that of the corresponding bulk material (fig. S2).

SEAL enables the fabrication of complex 3D microstructures that are otherwise challenging to generate, especially with materials such as PLGA. Our initial motivation for developing this process was based on our desire to create a PLGA microparticle platform to deliver timed pulses of antigens for essentially any vaccine in a single injection. To create these microparticles, fillable bases were molded using thermoplastic polymers and transferred to a glass slide to expose the empty particle core (Fig. 2A). Particle cores were filled with a model drug solution by using a BioJet Ultra picoliter dispensing apparatus (BioDot, Irving, California) (Fig. 2B and movie S3), aligned with capping polymer lids, pressed together, and briefly heated to seal the particles (Fig. 2C). Images of these particles after each stage of the fabrication process are shown in Fig. 2, D to K.

**Fig. 2:**
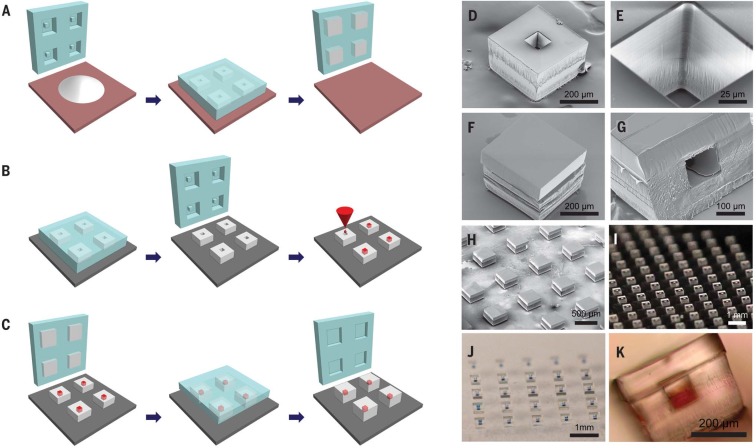
**SEAL-fabricated controlled-release microparticles.** Particles are fabricated by (**A**) heating and pressing polymer between a patterned PDMS base mold and a Teflon surface, (**B**) transferring these bases to a new substrate and filling them with a model drug of interest, then (**C**) aligning an array of particle caps with drug-filled bases and briefly applying a low amount of heat to sinter the two layers. SEM images of (**D**) a single particle, (**E**) the core of a particle, and (**F**) a sealed particle. (**G**) A cross section of a single particle and (**H**) an array of sealed particles. Optical images of (**I**) an array of bases, (**J**) an array of filled particles, and (**K**) a side view of a single filled particle.

These core-shell particles are potentially useful for biomedical applications because they are sufficiently small to be injected, can be created with fully biodegradable materials, and induce only a minimal foreign body reaction (fig. S3). Each particle weighs ~60 mg and has a theoretical loading of about 2%; however, much higher loading can be achieved by varying the wall thickness to yield larger cores (fig. S4). Unlike emulsion-based processes (17), loading and kinetics can be decoupled. These devices—or similar encapsulating structures—could be used for controlled release of a drug after an appropriate stimulus. Because our initial interest was in creating pulsatile release for single-injection immunizations, we studied the release kinetics of a model antigen from PLGA microparticles. In this approach, multiple particles with different compositions could be coinjected at the time of initial immunization, degrade over time, and release antigen in discrete pulses at time points that match typical vaccination schedules (Fig. 3A).

**Fig. 3:**
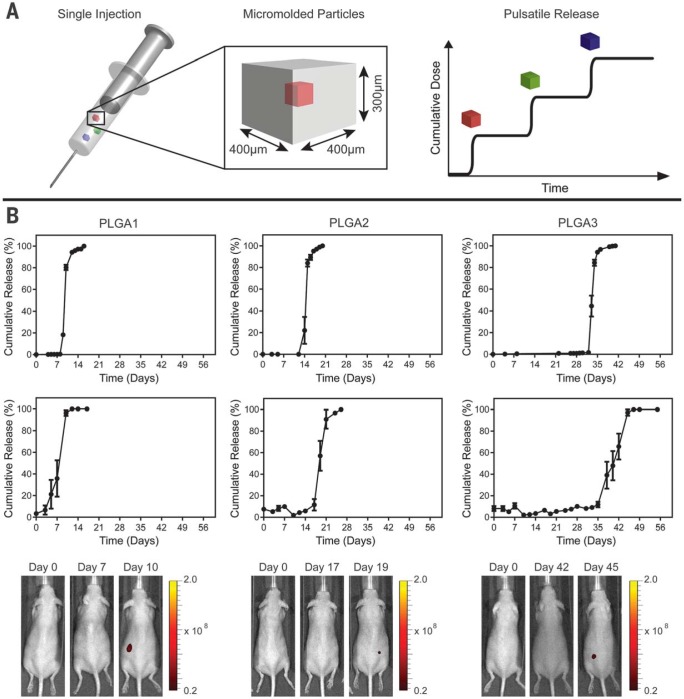
**Single-injection vaccination concept and release from SEAL-fabricated PLGA microparticles.** (**A**) Schematic of a syringe containing multiple micromolded particles sufficiently small to pass through an 18-gauge needle that each produce a discrete, delayed pulse of antigen release to mimic current bolus vaccination regimens. (**B**) In vitro and in vivo pulsatile release of encapsulated Alexa Fluor 680–labeled 10-kD dextran from SEAL-fabricated particles composed of PLGA1, PLGA2, and PLGA3, respectively, from left to right. The top row shows the in vitro cumulative release of fluorescently labeled dextran at 37°C (normalized average, *n* = 10 particles). Graphs in the second row depict the in vivo cumulative release (normalized average, *n* = 7 to 10 particles). Note that this yields a broader release curve even though each particle exhibits a sharp pulse because the onset of release can differ slightly in each animal. Error bars indicate the standard error of the mean. The third row shows representative images of mice collected with an in vivo imaging system after injection of a single SEAL-fabricated PLGA particle containing fluorescently labeled dextran.

We fabricated microparticles using three PLGA polymers with varying properties (table S1) and filled themicroparticles with fluorescently labeled dextran to observe release kinetics. Particles composed of PLGA1, PLGA2, or PLGA3 released in vitro at 10 ± 0, 15 ± 0, and 34 ± 1 days, respectively (Fig. 3B, first row). No measurable leakage was observed prior to release, indicating that this platformreleases its contents as a sharp pulse after degradation of the polymer barrier. A similar trend was observed when particles were subcutaneously injected into mice, as PLGA1, PLGA2, and PLGA3 particles released after 9 ± 2, 20 ± 1, and 41 ± 3 days in vivo, respectively (Fig. 3B, second row), as indicated by an ~50-fold increase in fluorescence upon release (fig. S5). Particles could also be lyophilized or frozen at –20°C without altering release kinetics (fig. S6). These results are especially exciting because they enable the production of various injectable microparticles that release their payloads in distinct, delayed bursts without prior leakage. Although a number of groups have created layered microparticles using microfluidic and other approaches (*18*, *19*), these methods produce particles with continuous release, whereas we show rapid drug release after a materialdependent delay.

We examined the compatibility of the SEAL process with biologics, including the trivalent inactivated polio vaccine (IPV), one of the most thermolabile vaccines in use today (*20*), and ovalbumin (OVA), a commonly studied model antigen. A formulation consisting of IPV, sucrose, monosodium glutamate, and magnesium chloride was dispensed into each PLGA1 particle core and dried spontaneously within seconds after filling. Particles were then exposed to the sealing process to determine if brief heating (~60 s at 42°C) had any effect on IPV stability. IPV Dantigenicity, a surrogate for the seroprotectionconferring antigen conformation used to evaluate clinical formulations (*21*), remained statistically similar before and after the sealing process, suggesting that the sealing step is relatively harmless to the encapsulated biologic (fig. S7). Type 1 IPV retained 98.1 ± 11.4% of D-antigenicity after filling, type 2 IPV retained 99.6 ± 7.8%, and type 3 IPV retained 103.8 ± 11.9% relative to filled, unsealed particles. Overall, the stability of the biologic during sealing in PLGA1 was much higher than what is typically reported for microsphere encapsulation with an emulsionbased process (*22*, *23*), likely due to the elimination of organic solvents, emulsification stressors, and washing, which can lead to substantial losses. After encapsulating and sealing IPV in PLGA microparticles, additional excipients may be needed to stabilize IPV against thermal and acidic pH stressors that it will encounter during longterm storage in the body (*24*, *25*). To achieve very long time points with this system, it may be necessary to use PLGA with different end groups, copolymer ratios, and/or molecular weights.Maintaining a low sealing temperature to minimize stress on the antigen can be achieved by using an ester end cap, which increases hydrophobicity and thus delays the onset of release.

OVA was then used to assess the potential of this platform as a single-injection vaccine. Mice received a single injection of 25 PLGA1 particles containing 10 μg of endotoxin-free OVA (EndoFit, Invivogen, San Diego, California) and 25 PLGA3 particles containing 10 mg of endotoxin-free OVA, for a total of 20 mg. The corresponding bolus controls were injected tomatch the time at which 25 coinjected microparticles of each PLGA1 and PLGA3 released fluorescent 10-kD dextran (fig. S8). Mice receiving OVA-filled core-shell particles achieved peak titers (20 ± 1 on a log_2_ scale) that were significantly higher than those in mice receiving two dose-matched boluses at 6 and 36 days (12 ± 4, P < 0.01). The experimental group was also statistically similar to mice receiving empty particles along with the two bolus injections (peak titer of 18 ± 5), suggesting that the PLGA particles themselves have an adjuvant effect, consistent with what has been previously reported (26). In addition, it appears that this adjuvant effect is sufficient to achieve approximately twofold dose sparing, because peak titers in the experimental group were statistically similar to those of two bolus injections containing double the antigen dose, indicating that this singleinjection strategy could potentially be used to replace multiple injections and enable the use of lower antigen doses. Longitudinal and peak antibody titers can be seen in Fig. 4, A and B, respectively. Additionally, storage did not affect the release or stability of OVA, as particles stored dry for 1 month at 4°C released similar amounts of enzyme-linked immunosorbent assay (ELISA)– reactive OVA compared to freshly prepared particles (Fig. 4C). These experiments demonstrate that one injection of core-shell particles can induce a long-termantibody response, outperform multiple time-matched injections, and achieve twofold dose sparing.

**Fig. 4:**
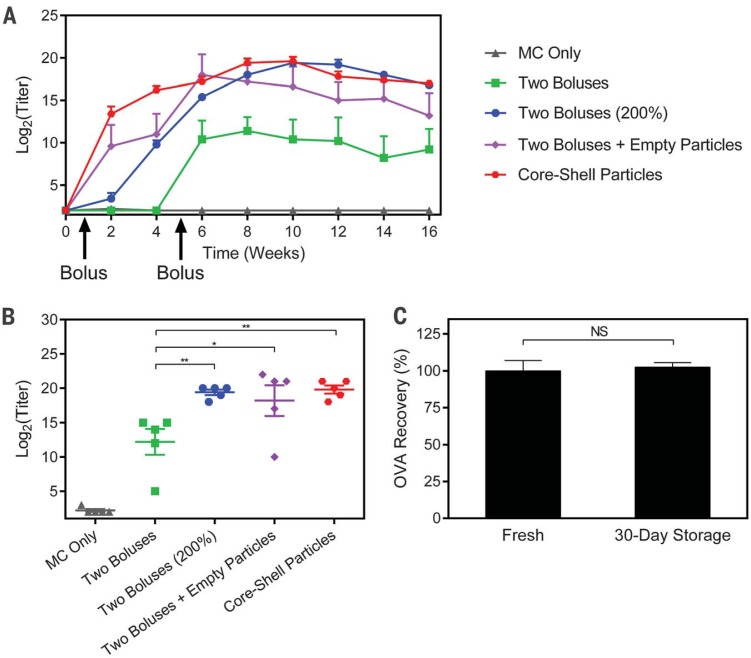
**Ovalbumin (OVA) vaccination and storage stability.** (**A**) Longitudinal geometricmean antibody titers and (**B**) peak antibody titers achieved by mice treated with a single injection of OVA-containing particles or two bolus injections of OVA in solution (*n* = 5 mice). All peak titer groups were significantly different from those of the control group receiving only methyl cellulose (MC; *P* < 0.001), but significance markers are not shown for clarity. All other differences are indicated by *P < 0.05 and **P < 0.01.These results show that a single injection of core-shell particles can induce an immune response that is not only noninferior to two dose-matched boluses, but also noninferior to two boluses with double the cumulative dose. (**C**) Core-shell particles stored desiccated for 30 days at 4°C release the same amount of ELISA-reactive OVA as freshly prepared particles (*n* = 4 particles). Error bars indicate standard error of the geometric mean in (A) and (B) and standard error of the mean in (C).

Overall, the ability to fabricate an internal compartment alone could enable a broad array of new applications with potential utility in biomedicine, sensing, actuation, and environmental monitoring. Stimuli-responsive materials could be used in environmental applications to detect harmful conditions and release a molecule that either serves as a warning signal, neutralizes the harmful agent, or protects a sensitive payload until it reaches the desired target for release (*27*, *28*). To examine this capability, we created a microscale pH sensor consisting of fluorescent dye encapsulated in a particle composed primarily of Eudragit FS 30 D (Evonik Industries, Essen, Germany), a pH-sensitive polymer that remains solid at low pH but rapidly dissolves at neutral pH. When subjected to a near-neutral environment (pH 7.5), these particles quickly released their contents, signaling that a change in pH had occurred (fig. S9, A to E). These particles were also able to protect a biologic from low-pH insult. OVA encapsulated in FS 30 D was released at neutral pH after incubation in a pH 1.2 solution at 37°C for 18 hours and retained 95 ± 8% of ELISA reactivity (fig. S9F). When fed to mice, these particles did not release their fluorescent payload in the low pH of the stomach; release occurred only after particles reached the desired target of the more neutral intestines (fig. S9, G to I). Other polymers that demonstrate the opposite behavior, such as Eudragit E PO (Evonik Industries), could be used in parallel for precise regulation of environmental conditions. We also demonstrate the ability of SEAL to create 3D microfluidic channels with a cross-sectional area of 50 µm by 50 µm using a three-layered PLGA structure that was embedded in PDMS and removed via dissolution in organic solvent (fig. S10, A to C). The resulting structure was a vertically serpentine hollow channel in PDMS that was subsequently bonded to glass and subjected to flow (fig. S10, D and E, and movie S4). These channels are optically transparent and smaller than the minimum reliable resolution produced by 3D printing, which has been reported to be 60 µmby 108 µmor 200 µm (*29*, *30*), while avoiding the bonding issues associated with multiple PDMS layers (*31*).

## Supplementary Material

Fabrication of fillable microparticles and other complex 3D microstructures

## SUPPLEMENTARY MATERIALS

www.sciencemag.org/content/357/6356/1138/suppl/DC1

Movies S1 to S4

Reference (32)
