# Supplementary material for: Fabrication of fillable microparticles and other complex 3D microstructures
Source: Science. 2017 Sep 15;359:1138–42. doi: 10.1126/science.aaf7447 (PMC6510330; doi:10.1126/science.aaf7447)
Supplement: Fabrication of fillable microparticles and other complex 3D microstructures [file Science-357-1138-s001.pdf]

www.sciencemag.org/content/357/6356/1138/suppl/DC1

Supplementary Materials for

Fabrication of fillable microparticles and other complex 3D microstructures

Kevin J. McHugh,* Thanh D. Nguyen,* Allison R. Linehan, David Yang, Adam M. Behrens, Sviatlana Rose, Zachary L. Tochka, Stephany Y. Tzeng, James J. Norman, Aaron C. Anselmo, Xian Xu, Stephanie Tomasic, Matthew A. Taylor, Jennifer Lu, Rohiverth Guarecuco, Robert Langer,† Ana Jaklenec†

*These authors contributed equally to this work.
†Corresponding author. Email: rlanger@mit.edu (R.L.); jaklenec@mit.edu (A.J.)

Published 15 September 2017, *Science* **357**, 1138 (2017)
DOI: 10.1126/science.aaf7447

This PDF file includes:

Materials and Methods
Figs. S1 to S10
Table S1
References
Captions for Movies S1 to S4

Other Supplementary Materials for this manuscript include the following:
(available at www.sciencemag.org/content/357/6356/1138/suppl/DC1)

Movies S1 to S4

Materials and Methods

Micromold Fabrication

Silicon wafers were patterned with microscale features to create master molds and then replicated in polydimethylsiloxane (PDMS) using soft lithography (Fig. S1). Photomasks with patterns corresponding to each layer in the StampEd Assembly of polymer Layers (SEAL) process were created using Layout Editor (Juspertor, Unterhaching, Germany) and made in-house or by Front Range Photomask (Palmer Lake, CO). A 3 µm-thick silicon dioxide layer was then deposited on a 150 mm silicon wafer using plasma-enhanced chemical vapor deposition with a recipe of 50 sccm SiH_4 and 800 sccm N_2O for 56.4 sec at 255 W, 400°C, and 2.7 Torr. This wafer was then spin-coated with AZ 4260 photoresist (MicroChemicals, Ulm, Germany) at 1,000 RPM for 60 sec, baked at 95°C for 1 hr, and then exposed to ultraviolet light through a photomask for 20 sec using an EV620 mask aligner (Electronic Visions, Rockledge, FL). Next, exposed photoresist was removed by developing the wafer in MIF405 (MicroChemicals) for 3 min. An oxide etch was then performed using 90 sccm of CF_4, 30 sccm of CHF_3, and 120 sccm of He for 9 min at 850 W and 2.8 Torr to remove the oxide. For layers with multiple desired heights such as the microparticle bases, a second round of photoresist deposition, aligned exposure, development, and etching was performed. The wafer was then etched using a Haber-Bosch process in a Pegasus deep reactive ion etcher (Surface Technology System, Newport, Wales) using an etch recipe of 450 sccm SF_6 and 45 sccm O_2 for 7 sec at 2800 W and passivation recipe of 200 sccm C_4F_8 for 4.5 sec at 2000 W to etch down exposed silicon to the desired height for each layer. After etching, the wafer was cleaned by acetone, isopropanol, and oxygen plasma at 1000 W, 0.9 mTorr O_2 for 1 hr. The Pegasus was then used to deposit approximately 300 nm of C_4F_8 (200 sccm of C_4F_8 for 1 min at 2000 W, 25 mTorr). This polymer film acts like Teflon and limits adhesion to PDMS in subsequent steps. PDMS base and curing agent were mixed in a 7:1 ratio (Sylgard 184, Dow Corning, Midland, Michigan), poured onto the silicon master mold, and degassed under vacuum for 1 hr. A thin PDMS mold was then produced by attaching two cover slips to the end of a glass slide as spacers and pushing down against the silicon mold while curing at 75°C for 2 hr.

Polymer Micromolding and Transfer Using Soft Lithography

Poly(lactic-co-glycolic acid) (PLGA) films were created by solvent casting 20-60% w/v PLGA in acetone on Teflon-coated glass and passing them under a doctor blade between 300 and 800 µm in height depending on the polymer. These films were then further dried overnight on a 60°C hot plate to yield rigid films 50-100 µm thick. A small piece of this PLGA film was then placed between the PDMS mold and a Teflon film and compressed under a spring-loaded clamp in a 120°C oven under high vacuum for 2-24 hr depending on the polymer. After cooling, the clamp and Teflon film were removed and separated yielding PLGA entrapped in the patterned depressions of the PDMS mold. In some cases, a thin film of PLGA on the order of a few hundred nanometers was still present between the microstructures, but did not affect layer-by-layer assembly. To transfer the bottom layer of each structure (such as the microparticle base) to a different substrate, the PDMS mold containing polymer was clamped against a glass slide and heated at 120°C for 5 min before being cooled with dry ice and separated. These structures could then be separated from the glass slide using forceps or a razor blade.

Layer-by-Layer Alignment and Sintering

Multi-layer polymer structures were precisely aligned and sintered using the method described in the main text. Briefly, a photomask aligner was used to rotate and translate adjacent polymer layers until they were visibly aligned. Layers were then brought into contact and heated until they fused and sealed. Rarely, a particle exhibited immediate release after in vivo injection, likely due to handling damage or inadequate manual sealing, and was excluded from the data set. Moving forward, this process would greatly benefit from automation to improve yield. The current manual process relies on visual inspection of diffraction patterns (Movie S2), but could be automated using an optical sensor or interferometer to capture the resolution of diffraction patterns upon sintering (*25*). The other challenges associated with the heat-assisted microtransfer molding step in SEAL are the possibility that the polymer does not release from the mold or that a nanoscale film forms that connects otherwise discrete features (*32*). However, these challenges can be readily overcome by silanizing the PDMS molds with tricholoro(1H,1H,2H,2H-perfluorooctyl)silane to promote second-layer delamination, compressing the mold with sufficient force to eliminate the connective film, or by replacing PDMS with a fluorinated elastomer as used in the Particle Replication in Non-wetting Templates (PRINT) method (*12*). There is likely some limit to the geometries that can be obtained based on the mechanical strength of the material being molded.

Particle and Polymer Characterization

Molecular weights were determined using a Viscotek TDA 305 (Malvern Instruments, Malvern, United Kingdom) using tetrahydrofuran as the solvent against poly(methyl methacrylate) standards. Glass transition temperature was determined by running samples on a Perkin Elmer Diamond Differential Scanning Calorimeter. Optical images of microstructures were stitched together from multiple images to enable a better depth of focus. The interfaces between images are denoted by thin red lines. High resolution images of particles were collected using a scanning electron microscope (SEM). First, samples were coated with a thin layer of Au/Pd using a Hummer 6.2 Sputtering System (Anatech, Battle Creek, MI) and then imaged using a JSM-5600LV SEM (JEOL, Tokyo, Japan) with an acceleration voltage of 5 kV. The brightness and contrast of these images has been modified post-collection for consistency and optimal electronic and print viewing. Mechanical testing was performed using a Dynamic Mechanical Analyzer Q800 (TA Instruments) equipped with a tension clamp. Samples were mounted and a force ramp of 1 N/min was applied. Each sample type was replicated 5 times.

Particle Filling

A BioJet Ultra picoliter dispensing apparatus was used to fill compounds into the particle core. A 50 mg/ml solution of Alexa Fluor 488- or 680-labeled 10 kD dextran (Life Technologies, Carlsbad, CA) was used as a model drug for ease of tracking. Microparticle cores were filled with solution using multiple ten-drop cycles of 100-150 pl drops. The volume filled during each cycle could exceed the volume of the particle core due to rapid evaporation and convex meniscus formation.

In Vitro Release Kinetics

Sealed particles with an outer footprint of 400 x 400 x 300 µm and 100 x 100 x 100 µm core were filled with 300 ng of Alexa Fluor 488-labeled 10 kD dextran. Each particle was then placed into 300 µl of phosphate-buffered saline (PBS) in a lo-bind microcentrifuge tube (Eppendorf, Hamburg, Germany) and incubated on a shaker at 37°C. Release was then measured

every 1-4 days depending on particle composition by analyzing the supernatant fluorescence at 475/520 nm in technical duplicate with a Tecan Infinite M200 spectrophotometer (Männedorf, Switzerland). Results were quantified using a standard curve and normalized to total cumulative release (n=10). At each time point, supernatant was replaced with 300 µl of fresh PBS. The timing of release is reported as the day at which more than half of the total payload has been released.

In Vivo Release Kinetics

The same types of particles studied in vitro were also used to study in vivo kinetics in female SKH1-Elite mice (SKH1-Hr^{hr}, Charles River Laboratories, Wilmington, MA) a hairless, but immunocompetent strain. All procedures were approved prior to beginning in vivo experiments by the MIT Committee on Animal Care. Ten days prior to injection, mice were switched to an alfalfa-free purified rodent diet (Harlan Laboratories, Madison, WI) to reduce intestinal auto-fluorescence. Particles were sterilized prior to surgery using a 20 µl drop of 70% ethanol which dried in approximately 1 min. On the day of injection, mice were anesthetized using continuous inhalation of 3% isoflurane and had their injection site sterilized with ethanol. Particles filled with Alexa Fluor 680-labeled 10 kD dextran were then tip-loaded into a 18 Monoject filter needle (Covidien, Dublin, Ireland) in approximately 100 µl of 15 mg/ml of 4,000 cP methyl cellulose (Sigma Aldrich) used as a viscosity enhancer and injected subcutaneously into the rear flank of the animal.

Mice were imaged using a PerkinElmer Spectrum In Vivo Imaging System (IVIS, Hopkinton, MA) weekly, then more frequently when approaching the projected release time. Mice were imaged 3 times per week (n=7-10, 1-2 particles/animal). At each imaging session, mice were anesthetized using continuous inhalation of 3% isoflurane and placed on the heated imaging platform. Fluorescent images were then collected using 640/700 nm or 640/720 nm excitation/emission filter sets with an F-Stop setting of 2 and subject height of 1.5 cm in Living Image 4.4 software. Cumulative release was normalized to the maximum and minimum total fluorescence in the region of interest corresponding to a particular particle's complete release and background signal, respectively. Because fluorescence dropped after release due to biological clearance, values after the highest signal was achieved were set to 100% in Fig. 3B. Release timing was considered to be the day on which fluorescence achieved half of its final maximum value above background. For visualization purposes, images were prepared using 3x3 smoothing, a binning setting of 4, and reported as radiant efficiency ($[p/sec/cm^2/sr]/[\mu W/cm^2]$). Prior to release, particle-associated fluorescence was on the order of background autofluorescence, likely due to self-quenching and/or desiccation; however, the signal increased substantially upon release and spreading as depicted in Figure S5.

To determine the effect of particle number and polymer composition on release kinetics, this study was repeated with a co-injection of multiple particle types. In order to allow for the differentiation of each particle population, three different fluorophores were used. A mixture of 25 PLGA1 particles containing 10 µg of Alexa Fluor 594-labeled 10 kD dextran, 25 PLGA2 particles containing 10 µg of Alexa Fluor 647-labeled 10 kD dextran, and 25 PLGA4 particles containing 10 µg of Alexa Fluor 680-labeled 10kD dextran were co-injected with an 18 G Monoject filter needle in approximately 300 µl of 15 mg/ml of methyl cellulose used as a viscosity enhancer. Mice were imaged regularly at 570/620 nm, 640/680 nm, and 675/780 nm excitation/emission, which were found to be the most effective filter sets that provided a high signal while minimizing overlap from adjacent dyes. Release timing was reported as the day

between imaging sessions at which the fluorescence increased the most in the corresponding filter set.

Histology

SKH1-Elite mice were injected subcutaneously with PLGA3 particles containing 400 µg of Alexa Fluor 680-labeled 10 kD dextran to help identify the implant location after degradation. After 2, 4, or 8 weeks mice were euthanized and their skin and adjacent sub-dermal tissue was harvested, fixed in formalin-free fixative (Sigma Aldrich) for 24 hr, and transferred to 70% ethanol. Tissue was then embedded in paraffin, cut into 5 µm-thick tissue sections, stained with hematoxylin & eosin or Masson's trichrome stains, and imaged using an Aperio AT2 Slide Scanner (Leica Biosystems, Buffalo Grove, IL).

SEAL Compatibility with Sensitive Biomacromolecules

The inactivated polio vaccine (IPV) was used as a therapeutically relevant model for sensitive biomacromolecules since it is one of the least thermostable vaccines in widespread use today (*20*). IPV is rendered ineffective due to heat, solvent, or other environmental factors. IPV provided by the Statens Serum Institut (Copenhagen, Denmark) at approximately 10x clinical concentration was further concentrated using Amicon Ultra-0.5 ml Centrifugal Filters with a 100 kD cutoff (EMD Millipore, Billerica, MA). After centrifuging the sample for 10 min at 14,000 RCF, filters were inverted and centrifuged again for 2 min at 1,000 RCF to collect the concentrated stock. This stock was then desalted to remove formulation excipients using 2 ml ZEBA Spin Desalting Columns with a 40 kD cutoff (Thermo Fisher Scientific, Waltham, MA). Sample was loaded into the water-equilibrated desalting columns and centrifuged for 3 min at 1,000 RCF. This process was repeated a second time to further purify the stock. The concentrated, desalted stock was then combined 1:1 with a solution of 20% (w/v) sucrose, 17% (w/v) monosodium glutamate, and 17% (w/v) $MgCl_2$ in water. This solution was loaded into core-shell bases using the BioJet Ultra dispensing machine in two cycles, each consisting of ten 150 pl drops for a total volume of approximately 3 nl. Particles were then sealed with an offset cap using the aforementioned sealing process at 40°C for 3 min. Particles that were filled and capped or filled without capping were then placed in antigen dilution buffer (1% bovine serum albumin, 1% Triton X-100, 0.001% phenol red in PBS) and vortexed to recover the IPV. Samples were then diluted and analyzed using a D-antigen-specific IPV enzyme-linked immunosorbent assay (Statens Serum Institut) using the protocol previously reported (*21*).

Ovalbumin (OVA) Vaccination

SKH1-Elite mice were injected at two sites in the rear flank with a mixture of 25 PLGA1 particles containing 10 µg of endotoxin-free OVA and 25 PLGA3 particles containing 10 µg of endotoxin-free OVA (20 µg total) via an 18G filter needle in approximately 300 µl of 15 mg/ml methyl cellulose. Based on in vivo fluorescent dextran release experiments in which this quantity of mixed-population particles was injected, PLGA1 and PLGA3 particles were expected to release the largest amount of antigen 6 and 36 days after injection, respectively (Fig. S7); therefore, bolus controls were administered at these time points. Control mice received either two dose-matched injections of OVA at 6 and 36 days (20 µg total), two double-dose injections of OVA at 6 and 36 days (40 µg total), or an injection of empty (i.e. unfilled) particles on day 0 followed by 10 µg OVA injections at 6 and 36 days (20 µg total). For the group receiving 25 empty PLGA1 and 25 empty PLGA3 particles, these were injected on day 0 and then bolus OVA

injections were administered at 6 and 36 days. The soluble OVA used in all control groups was also aliquoted by the BioJet dispensing system.

OVA-specific total IgG antibody titers were assessed using a blood serum enzyme-linked immunosorbent assay (ELISA) and reported as $\log_2$ values. Mice were bled every two weeks from the submandibular vein. Serum was then isolated from whole blood by centrifuging samples at 2,000 RCF for 10 min at 4°C and stored at -20°C. 50 µl of 20 µg/ml OVA in 100 mM carbonate-bicarbonate buffer (pH 9.6) was added to 96-well plates and incubated overnight at 4°C. Plates were then washed with 0.05% Tween 20 in PBS and blocked for 1 hr at 37°C with 5% non-fat dry milk resuspended in 0.05% Tween 20 in PBS. Blocking buffer was then replaced with two-fold serial dilutions of serum diluted in blocking buffer and incubated for 2 hr at 37°C. The solution was then aspirated, washed, and replaced with a 1:1000 dilution of monoclonal goat anti-mouse IgG HRP-conjugated antibody in blocking buffer. After 1 hr incubation at 37°C, wells were washed again and 100 µl of OPD peroxidase substrate was added to the wells according to the manufacturer's instructions. The reaction was then stopped approximately 30 min later by adding 1 M H_2SO_4 before measuring absorbance at 490 nm using a Tecan Infinite M200 spectrophotometer. Titers are reported as the most dilute serum sample that yielded an absorbance reading ≥ 2-fold higher than serum from untreated mice at the same dilution. Statistical comparisons for peak titers were performed in GraphPad Prism (GraphPad Software, La Jolla, CA) using an ordinary one-way analysis of variance (ANOVA) with a Tukey multiple comparison test.

To assess "on-shelf" stability, PLGA1 particles were filled with 300 ng of EndoFit OVA and sealed. Particles were then either immediately incubated in PBS at 37°C on an orbital shaker or stored desiccated at 4°C for 30 days before incubation. After 12 days of incubation, the supernatant was collected, diluted, and assessed using a Chicken Egg Ovalbumin ELISA (Alpha Diagnostics International, San Antonio, TX) according to the manufacturer’s instructions.

pH Sensitive Microparticles

Films of FS 30 D were created by mixing 30 g of FS 30 D aqueous dispersion (30% solids) and 1 g of triethyl citrate for 45 min at 300 RPM at room temperature. This mixture was cast on a 150 mm polystyrene petri dish and dried at 80°C overnight. FS 30 D films were then removed and molded into particle caps and bases in a 120°C oven for 2 hr. Particles were filled with Alexa Fluor 488-labeled 10 kD dextran were incubated in 1 ml of either simulated gastric fluid (SGF, pH 1.2) or simulated intestinal fluid (SIF, pH 7.5), and placed in an Eppendorf thermomixer at 37°C and 1000 RPM. Release was measured at 1, 2, 4, and 7 hr by analyzing the fluorescence of the supernatant as described above.

To test the ability of FS 30 D particles to protect OVA from degradation at low pH in vitro, particles filled with OVA were suspended with 1 ml SGF, and rotated (24 RPM) at 37°C for 18 hr and compared to uncapped OVA particles that allowed for its rapid dissolution in SGF. After 18 hr, particles were transferred to 1 ml of SIF and rotated (24 RPM) for an additional 2 hr at 37°C to release the encapsulated OVA. All samples were neutralized in SIF, diluted in sample diluent, and run on the Chicken Egg Ovalbumin ELISA according the manufacturer’s instructions. Experimental samples were compared to a control group of filled particles that were only incubated in SIF for 2 hr.

In vivo release experiments were peformed in SKH1-Elite mice. FS 30 D were filled with 100 ng of Alexa Fluor 750 NHS Ester (Thermo Fisher Scientific), which was chosen due to its high wavelength (e.g. low autofluorescence and tissue absorption) and statistically similar

fluorescence intensity for pH 2-8 in vitro as analyzed using one-way ANOVA ($p=0.23$). Particles were then aligned, sealed at 40°C, and removed from the slide. Mice then received either ten particles containing Alexa Fluor 750 or an equivalent amount of dye in 200 µl of water via gavage. Mice were imaged longitudinally using IVIS at 745/800 nm ex/em while anesthetized by continuous inhalation of 3% isoflurane to observe the location and intensity of fluorescence over time. After 7 hours, mice were euthanized using carbon dioxide inhalation and had their gastrointestinal tracts removed for end-point IVIS imaging.

Microfluidic Devices

Silicon wafers 100 mm in diameter were coated with 50 µm or 100 µm of SU-8 3050 (Microchem) by spinning at a speed of 1600 or 800 RPM for 60 sec, respectively. The wafer was then baked on a hot plate at 60°C for 5 min and 95°C for a minimum of 15 min until excess solvent had evaporated. The wafer was then exposed using a Karl Suss MA-4 through a photomask patterned with 400 x 50 µm and 50 x 50 µm rectangles that would become the first, second, and third layer molds. The wafer was then post-baked at 60°C for 1 min and 95°C for 5 min. Soluble photoresist was removed from the wafer using propylene glycol monomethyl ether acetate and finished by soaking in isopropanol. These molds were treated in a chamber of tricholoro(1H,1H,2H,2H-perfluorooctyl)silane for 1 hr and used to generate PDMS inverse molds. PLGA structures were then made using these molds and sealed using the methods described above. The dimensions of the resulting three-layer microstructure based on photomask and SU-8 parameters can be seen in Figure S10A. Sylgard 184 was then cast around the PLGA and cross-linked at 37°C for 48 hr. After curing, PDMS was soaked in dimethylformamide for 2 hr to remove PLGA leaving behind an empty channel. The bottom channel interface and a glass slide were treated with air plasma using a Harrick Plasma Cleaner PDC-091-HP (Ithica, NY) for 1 minute on high power at 400 mTorr and pressed together to form a bond. Alexa Fluor 488-labeled 10 kD dextran was then suspended in water at 1 mg/ml and manually injected into a 50 µm microfluidic device using a 500 µl syringe and 30-gauge needle.

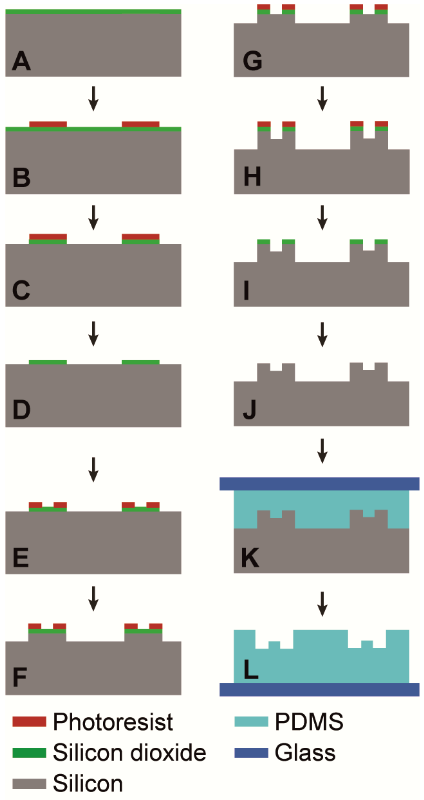

Fig. S1. Master and PDMS mold fabrication for a two-height layer.

Micropatterned PDMS molds were generated using the following steps: (A) chemical vapor deposition of silicon dioxide, (B) photoresist spin-coating and exposure, (C) unprotected oxide etch, (D) photoresist removal with oxygen plasma, (E) secondary photoresist spin-coating, aligned exposure, and development (F) primary deep reactive ion etch, (G) secondary unprotected oxide etch, (H) secondary deep reactive ion etch, (I) oxygen plasma treatment, (J) final oxide removal, (K) silicon mold fluorination and PDMS casting, and (L) PDMS curing and delamination.

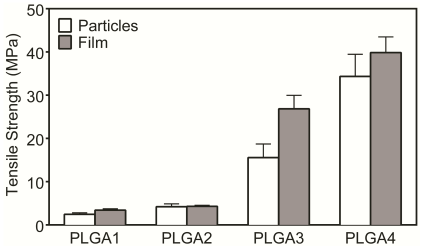

Fig. S2. Mechanical characterization of SEAL-bonded layers.

The adhesion strength between the caps and bases of particles was statistically similar to the tensile strength of the bulk material for PLGA1 and PLGA2, and PLGA4, but slightly lower ($p=0.03$) for PLGA3, $n=5$. Error bars indicate standard error of the mean.

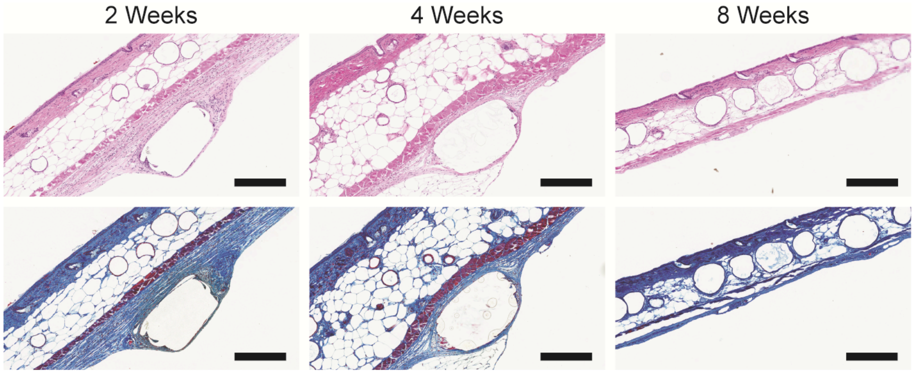

Fig. S3. Representative histological staining of the tissue surrounding a PLGA3 microparticle 2, 4, and 8 weeks after subcutaneous injection.

Hematoxylin & eosin and Masson's trichrome staining indicates only a minor foreign body reaction around the PLGA3 particle with relatively few macrophages present at the tissue/biomaterial interface, thin fibrous capsule formation, and little evidence of particle injection by 8 weeks. The particle also appears to largely retain its shape prior to release. All scale bars indicate 300 µm.

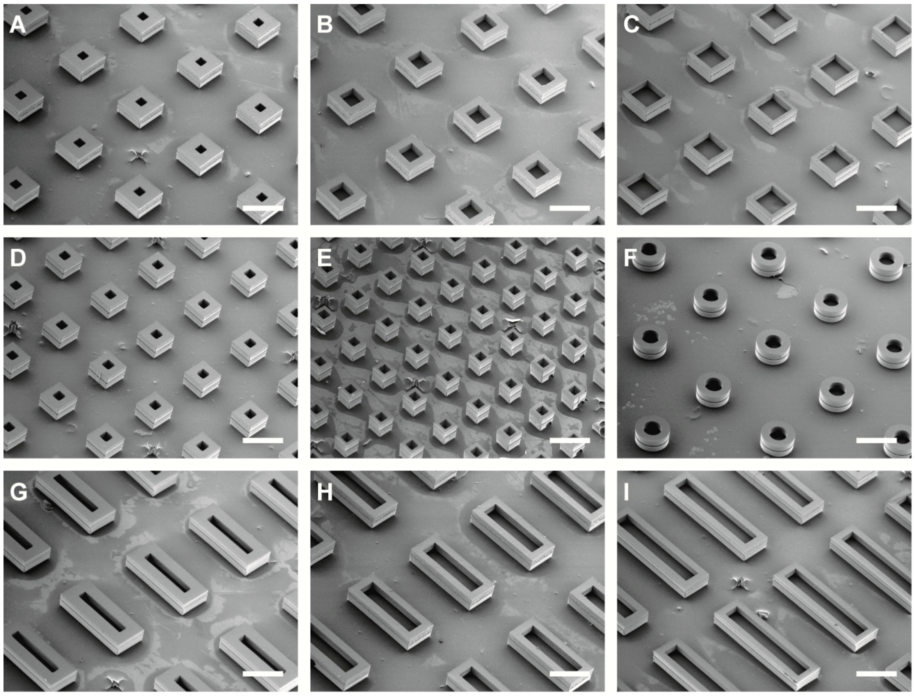

Figure S4. SEM images of alternative particle base geometries.

The particle presented in this paper possesses a theoretical loading of approximately 1 µg (2% w/w); however, other geometries with higher loading capacities are also readily achievable by altering the base and cap geometries including cylinders and long rectangular prisms that could be align with the long axis of the needle. Here we fabricated PLGA1 particles with a variety of internal core and external footprint dimensions resulting in theoretical loading of (A) 2%, (B) 8%, (C) 19%, (D) 4%, (E) 13%, (F) 8%, (G) 5%, (H) 20%, and (I) 22%. All scale bars indicate 500 µm.

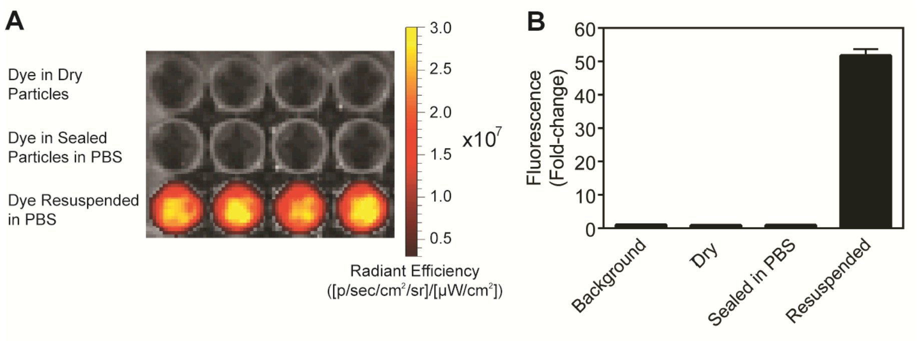

Fig. S5. Fluorescence from Alexa Fluor 647-labeled 10 kD dextran in particle cores.

(A) IVIS image and (B) graph displaying the increase in signal from fluorescent dextran after release from micromolded particles (excitation/emission: 640/720 nm), n=4. Error bars represent standard error of the mean.

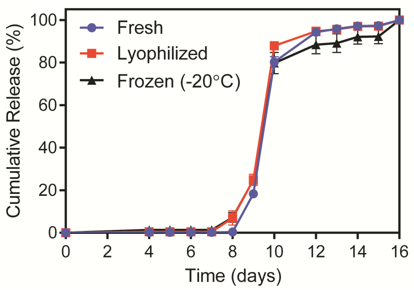

Fig. S6. Release kinetics are unaltered by storage processes.

PLGA1 particles filled with Alexa Fluor 488-labeled 10 kD dextran did not show any significant difference in release kinetics when used immediately after sealing, after freezing at -20°C or after lyophilization, n=10. Error bars represent standard error of the mean.

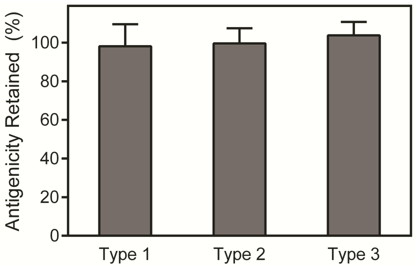

Fig. S7. IPV stability after encapsulation using SEAL.

The brief heating process associated with SEAL did not result in any significant change in trivalent IPV D-antigenicity as determined by monoclonal ELISA. Data normalized to filled, unsealed particles containing IPV, n=3-4. Error bars indicate standard error of the mean.

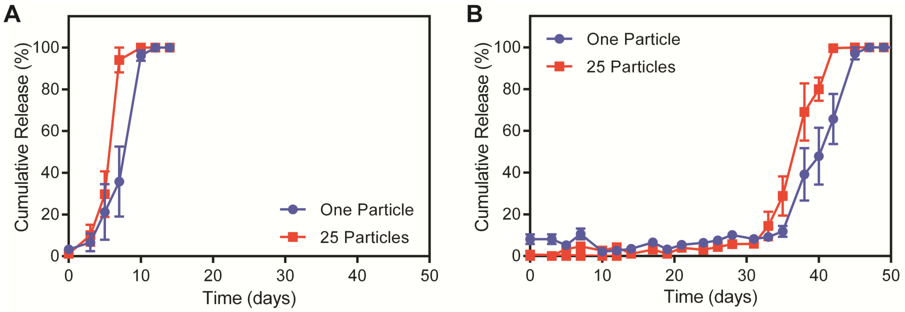

Fig. S8. In vivo release kinetics from mixed particle populations.
A mixture of 25 PLGA1 particles containing 10 µg of Alexa Fluor 594-labeled 10 kD dextran, 25 PLGA3 particles containing 10 µg of Alexa Fluor 647-labeled 10 kD dextran, and 25 PLGA4 particles containing 10 µg of Alexa Fluor 680-labeled 10kD dextran were co-injected into SKH1-Elite mice, imaged regularly to observe their respective release kinetics, and compared to a single particle of each particle type. The in vivo release kinetics of both (A) PLGA1 and (B) PLGA3 were slightly accelerated by the increase in the number of particles or the other populations of polymers compared to a single particle, n=5-7. Error bars indicate standard error of the mean.

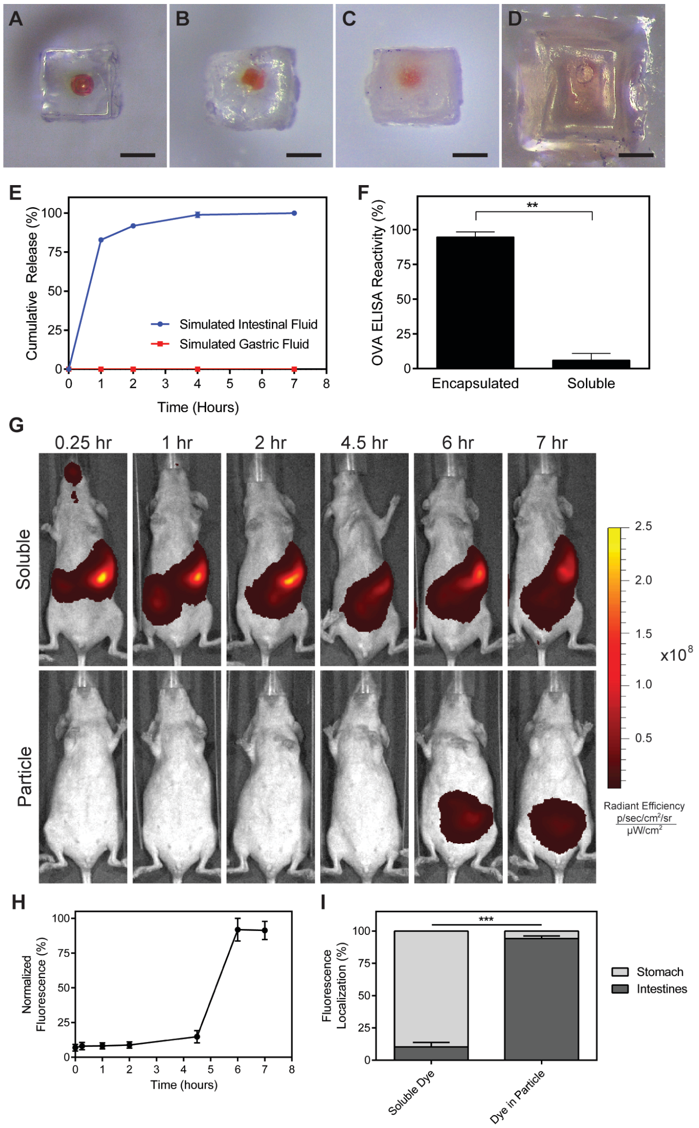

Fig. S9. pH-responsive particles for environmentally-triggered release and protection. Optical images of SEAL-fabricated microparticles composed of Eudragit FS 30 D (A) before incubation. These particles remain stable (B) after 7 hr and (C) 18 hr in simulated gastric fluid (SGF, pH 1.2), but begin releasing (D) after 30 min in simulated intestinal fluid (SIF, pH 7.5). Scale bars indicate 200 µm. (E) These particles rapidly released a majority of their encapsulated fluorescently labeled dextran within 1 hr in SIF, but demonstrated no release in SGF even after 7 hr, n=3. (F) FS 30 D particles were also able to protect OVA from SGF for 18 hr, n=5. (G) Representative longitudinal fluorescence imaging (745/800 nm ex/em) of mice receiving Alexa Fluor 750 in solution or encapsulated in FS 30 D particles administered via gavage. Fluorescence is immediately visible in the stomach of mice treated with soluble dye, but only appears lower in the body, likely the intestines, after 6 hr in the group receiving particles. (H) Quantification of fluorescence in mice treated with dye in particles showing a substantial increase at 6 hr, n=3. (I) Localization of fluorescence in the explanted gastrointestinal tracts of mice treated with soluble or encapsulated dye, n=3 confirms localization of dye from particles in the intestines. Error bars indicate standard error of the mean. ** $p<0.01$, *** $p<0.001$.

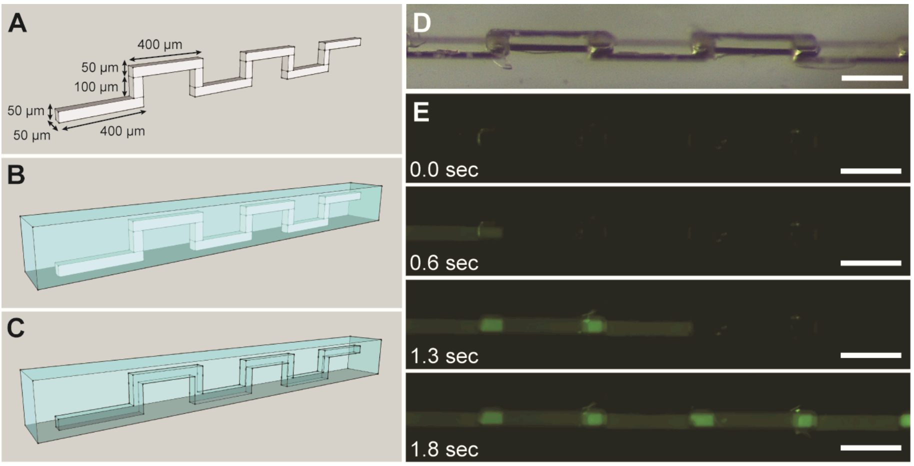

Fig. S10. Microfluidic device created using the SEAL method.

(A) Three layers of PLGA were assembled using the SEAL method. (B) The polymeric structure was then covered in PDMS and (C) dissolved in dimethylformamide after PDMS cross-linking leaving behind a 3D microfluidic channel. (D) An optical image of the channel on an angle at 8x magnification and (E) a top-down fluorescent images of a 1 mg/ml Alexa Fluor 488-labeled 10 kD dextran solution as the channel is filled manually using a 30G needle and 500 µl syringe. Scale bars indicate 200 µm.

Table S1. Melting temperatures of polymers used with SEAL.

Polymer Name	Source Material	Molecular Weight (kD)	Lactic Acid: Glycolic Acid	End Group	Polydispersity	Glass Transition Temperature (°C)
PLGA1	Evonik Resomer 502H	19	50:50	Carboxylic Acid	1.79	42
PLGA2	PolySciTech AP045	53	50:50	Carboxylic Acid	1.98	43
PLGA3	Evonik Resomer 505	75	50:50	Ester	1.85	48
PLGA4	Evonik Resomer 858S	214	85:15	Carboxylic Acid	1.76	53

Movie S1. The SEAL process. The StampEd Assembly of polymer Layers, or "SEAL" method, combines computer-chip manufacturing technology with a new layer-by-layer assembling method to create an array of core-shell polymer particles. First, the polymer of choice, PLGA in this example, is melt pressed using a prefabricated silicone mold. The mold with the particles is then transferred to another substrate where it is peeled off, leaving behind an array of polymer base particles. These are then filled with any drug or vaccine using an ink jet piezoelectric nozzle and then dried. To seal the filled particles, a cap mold is aligned, sealed with the base, and delaminated. The resulting array of core-shell particles are then removed from the base and stored until use.

Movie S2. Visual inspection of diffraction patterns during sintering. Adjacent microstructure layers, such as the particle base and cap shown here, are placed into contact resulting in a diffraction pattern due to a small air gap. Layers begin to fuse as the polymer is heated above its glass transition temperature thereby eliminating the small air gap and causing the diffraction pattern to resolve.

Movie S3. Particle ink jet filling. Micromolded particle bases with a 100 x 100 µm core and 400 x 400 x 200 µm external dimension are filled prior to sealing using a BioJet Ultra ink jet piezoelectric nozzle that can rapidly dispense picoliter volumes of model drug, such as the blue dye seen here, into the particle core. Filling shown at the default of 4 drops/sec for the purposes of visualization, but the equipment is capable of achieving rates up to 50 drops/sec.

Movie S4. Flow through a 3D microfluidic channel produced using SEAL. A solution of 1 mg/ml Alexa Fluor 488-labeled 10 kD dextran was manually injected into a 50 µm microfluidic device using a 500 µl syringe and 30-gauge needle. The solution is seen filling the channel both laterally and vertically through the continuous 3D serpentine channel.

References and Notes

1. Q. Xu, Y. Lv, C. Dong, T. S. Sreeprased, A. Tian, H. Zhang, Y. Tang, Z. Yu, N. Li, Three-dimensional micro/nanoscale architectures: Fabrication and applications. *Nanoscale* **7**, 10883–10895 (2015). doi:10.1039/C5NR02048D Medline

2. A. E. Jakus, E. B. Secor, A. L. Rutz, S. W. Jordan, M. C. Hersam, R. N. Shah, Three-dimensional printing of high-content graphene scaffolds for electronic and biomedical applications. *ACS Nano* **9**, 4636–4648 (2015). doi:10.1021/acsnano.5b01179 Medline

3. D. A. LaVan, T. McGuire, R. Langer, Small-scale systems for in vivo drug delivery. *Nat. Biotechnol.* **21**, 1184–1191 (2003). doi:10.1038/nbt876 Medline

4. N. Jones, Science in three dimensions: The print revolution. *Nature* **487**, 22–23 (2012). doi:10.1038/487022a Medline

5. J. A. Lewis, Novel inks for direct writing in three dimensions. *Digital Fabrication 2005, Final Program and Proceedings*, 142-142 (2005).

6. J. W. Stansbury, M. J. Idacavage, 3D printing with polymers: Challenges among expanding options and opportunities. *Dent. Mater.* **32**, 54–64 (2016). doi:10.1016/j.dental.2015.09.018 Medline

7. H. N. Chia, B. M. Wu, Recent advances in 3D printing of biomaterials. *J. Biol. Eng.* **9**, 4 (2015). doi:10.1186/s13036-015-0001-4 Medline

8. M. S. Mannoor, Z. Jiang, T. James, Y. L. Kong, K. A. Malatesta, W. O. Soboyejo, N. Verma, D. H. Gracias, M. C. McAlpine, 3D printed bionic ears. *Nano Lett.* **13**, 2634–2639 (2013). doi:10.1021/nl4007744 Medline

9. K. Sun, T.-S. Wei, B. Y. Ahn, J. Y. Seo, S. J. Dillon, J. A. Lewis, 3D printing of interdigitated Li-ion microbattery architectures. *Adv. Mater.* **25**, 4539–4543 (2013). doi:10.1002/adma.201301036 Medline

10. J. R. Tumbleston, D. Shirvanyants, N. Ermoshkin, R. Janusziewicz, A. R. Johnson, D. Kelly, K. Chen, R. Pinschmidt, J. P. Rolland, A. Ermoshkin, E. T. Samulski, J. M. DeSimone, Continuous liquid interface production of 3D objects. *Science* **347**, 1349–1352 (2015). doi:10.1126/science.aaa2397 Medline

11. H. N. Chia, B. M. Wu, High-resolution direct 3D printed PLGA scaffolds: Print and shrink. *Biofabrication* **7**, 015002 (2014). doi:10.1088/1758-5090/7/1/015002 Medline

12. J. P. Rolland, B. W. Maynor, L. E. Euliss, A. E. Exner, G. M. Denison, J. M. DeSimone, Direct fabrication and harvesting of monodisperse, shape-specific nanobiomaterials. *J. Am. Chem. Soc.* **127**, 10096–10100 (2005). doi:10.1021/ja051977c Medline

13. J. L. Perry, K. P. Herlihy, M. E. Napier, J. M. Desimone, PRINT: A novel platform toward shape and size specific nanoparticle theranostics. *Acc. Chem. Res.* **44**, 990–998 (2011). doi:10.1021/ar2000315 Medline

14. D. A. Klosterman *et al.*, Structural composites via Laminated Object Manufacturing (LOM). *Sol Freeform Fabric*, 105-115 (1996).

15. N. Ferrell, J. Woodard, D. Hansford, Fabrication of polymer microstructures for MEMS: Sacrificial layer micromolding and patterned substrate micromolding. *Biomed. Microdevices* **9**, 815–821 (2007). doi:10.1007/s10544-007-9094-y Medline

16. G. Vozzi, C. Flaim, A. Ahluwalia, S. Bhatia, Fabrication of PLGA scaffolds using soft lithography and microsyringe deposition. *Biomaterials* **24**, 2533–2540 (2003). doi:10.1016/S0142-9612(03)00052-8 Medline

17. F. Y. Han, K. J. Thurecht, A. K. Whittaker, M. T. Smith, Bioerodable PLGA-Based Microparticles for Producing Sustained-Release Drug Formulations and Strategies for Improving Drug Loading. *Front. Pharmacol.* **7**, 185 (2016). doi:10.3389/fphar.2016.00185 Medline

18. R. Vasiliauskas, D. Liu, S. Cito, H. Zhang, M.-A. Shahbazi, T. Sikanen, L. Mazutis, H. A. Santos, Simple microfluidic approach to fabricate monodisperse hollow microparticles for multidrug delivery. *ACS Appl. Mater. Interfaces* **7**, 14822–14832 (2015). doi:10.1021/acsami.5b04824 Medline

19. S. Yuan, F. Lei, Z. Liu, Q. Tong, T. Si, R. X. Xu, Coaxial Electrospray of Curcumin-Loaded Microparticles for Sustained Drug Release. *PLOS ONE* **10**, e0132609 (2015). doi:10.1371/journal.pone.0132609 Medline

20. D. Chen, D. Kristensen, Opportunities and challenges of developing thermostable vaccines. *Expert Rev. Vaccines* **8**, 547–557 (2009). doi:10.1586/erv.09.20 Medline

21. C. Singer, F. Knauert, G. Bushar, M. Klutch, R. Lundquist, G. V. Quinnan Jr., Quantitation of poliovirus antigens in inactivated viral vaccines by enzyme-linked immunosorbent assay using animal sera and monoclonal antibodies. *J. Biol. Stand.* **17**, 137–150 (1989). doi:10.1016/0092-1157(89)90004-8 Medline

22. J. L. Cleland, A. J. Jones, Stable formulations of recombinant human growth hormone and interferon-gamma for microencapsulation in biodegradable microspheres. *Pharm. Res.* **13**, 1464–1475 (1996). doi:10.1023/A:1016063109373 Medline

23. H. Sah, Protein instability toward organic solvent/water emulsification: Implications for protein microencapsulation into microspheres. *PDA J. Pharm. Sci. Technol.* **53**, 3–10 (1999). Medline

24. S. Y. Tzeng, R. Guarecuco, K. J. McHugh, S. Rose, E. M. Rosenberg, Y. Zeng, R. Langer, A. Jaklenec, Thermostabilization of inactivated polio vaccine in PLGA-based microspheres for pulsatile release. *J. Control. Release* **233**, 101–113 (2016). doi:10.1016/j.jconrel.2016.05.012 Medline

25. A. Giteau, M. C. Venier-Julienne, A. Aubert-Pouëssel, J. P. Benoit, How to achieve sustained and complete protein release from PLGA-based microparticles? *Int. J. Pharm.* **350**, 14–26 (2008). doi:10.1016/j.ijpharm.2007.11.012 Medline

26. D. T. O'Hagan, D. Rahman, J. P. McGee, H. Jeffery, M. C. Davies, P. Williams, S. S. Davis, S. J. Challacombe, Biodegradable microparticles as controlled release antigen delivery systems. *Immunology* **73**, 239–242 (1991). Medline

27. F. Lefèvre, A. Chalifour, L. Yu, V. Chodavarapu, P. Juneau, R. Izquierdo, Algal fluorescence sensor integrated into a microfluidic chip for water pollutant detection. *Lab Chip* **12**, 787–793 (2012). doi:10.1039/C2LC20998E Medline

28. Y. J. Lee, P. V. Braun, Tunable inverse opal hydrogel pH sensors. *Adv. Mater.* **15**, 563–566 (2003). doi:10.1002/adma.200304588

29. N. Bhattacharjee, A. Urrios, S. Kang, A. Folch, The upcoming 3D-printing revolution in microfluidics. *Lab Chip* **16**, 1720–1742 (2016). doi:10.1039/C6LC00163G Medline

30. S. Waheed, J. M. Cabot, N. P. Macdonald, T. Lewis, R. M. Guijt, B. Paull, M. C. Breadmore, 3D printed microfluidic devices: Enablers and barriers. *Lab Chip* **16**, 1993–2013 (2016). doi:10.1039/C6LC00284F Medline

31. M. A. Eddings, M. A. Johnson, B. K. Gale, Determining the optimal PDMS-PDMS bonding technique for microfluidic devices. *J. Micromech. Microeng.* **18**, 067001 (2008). doi:10.1088/0960-1317/18/6/067001

32. X. M. Zhao, Y. N. Xia, G. M. Whitesides, Fabrication of three-dimensional micro-structures: Microtransfer molding. *Adv. Mater.* **8**, 837–840 (1996). doi:10.1002/adma.19960081016
